# Anévrysme ventriculaire gauche et communication interventriculaire compliquant un infarctus du myocarde

**DOI:** 10.11604/pamj.2014.17.321.3123

**Published:** 2014-04-28

**Authors:** Mohammed Belkhadir, Younes MoutakiAllah, Zainab Raissouni, Abdessamad Abdou, Mehdi Bamous, Fouad Nya, Noureddine Atmani, Mahdi Ait Houssa, Youssef El Bekkali, Abdellatif Boulahya

**Affiliations:** 1 Service de Chirurgie Cardiovasculaire Hôpital Militaire d'Instruction, Mohammed V- Université Mohamed V Souissi, Rabat, Maroc

**Keywords:** Infarctus du myocarde, anévrysme ventriculaire gauche, communication inter ventriculaire, Myocardial infarction, left ventricular aneurysm, interventricular communication

## Abstract

L'association d'une communication interventriculaire post infarctus du myocarde et d'un anévrysme du ventricule gauche chez un même patient est extrêmement rare et survient habituellement durant la première semaine qui suit un infarctus du myocarde. Nous rapportons le cas insolite d'un patient âgé de 63 ans, admis pour choc cardiogénique en rapport avec une communication inter ventriculaire apicale et un anévrysme ventriculaire gauche causés par un infarctus du myocarde antérieur. La correction chirurgicale a consisté en une fermeture du défect septal par un patch en dacron via une ventriculotomie gauche associée à une anévrysectomie et un mono pontage coronaire. Cette observation illustre d'une part la rareté de l'association communication inter ventriculaire-anévrysme ventriculaire gauche post infarctus du myocarde, et d'autre part l'efficacité du traitement chirurgical qui reste la seule option salvatrice pour cette pathologie.

## Introduction

Les complications mécaniques de l'infarctus du myocarde (IDM) sont redoutables, et surviennent volontiers suite à un IDM transmural. La communication inter ventriculaire (CIV) post infarctus survient habituellement dans la semaine qui suit l’épisode inaugural. Son incidence globale est de 1 à 3%; elle est généralement associée à une mortalité élevée. Les anévrysmes ventriculaires gauches ont une incidence de 3. 5 à 38%; leur survenue a été décrite pour la première fois en 1757 par John Hunter. La présence simultanée d'une CIV et d'un anévrysme du ventricule gauche (VG) chez un même patient est extrêmement rare et survient habituellement durant la première semaine qui suit l'IDM. La chirurgie reste la seule option thérapeutique valable.

## Patient et observation

Mr E. K, patient âgé de 63 ans, tabagique chronique et diabétique type 2, a été admis aux urgences de l'hôpital militaire de Rabat dans un tableau de choc cardiogénique réfractaire avec une hypotension artérielle systémique, des marbrures au niveau des extrémités, un pouls filant avec une dyspnée stade III de la NYHA et un angor instable, cinq semaines après la survenue d'un IDM antérieur non thrombolysé; associé à un souffle holosystolique au bord gauche du sternum irradiant à l'apex et des râles crépitants au niveau des deux champs pulmonaires. Devant l'instabilité hémodynamique du patient, il a été admis en unité de soins intensifs avec pose d'une voie veineuse centrale jugulaire et d'un cathéter de pression artérielle invasive.

**Figure 1:**
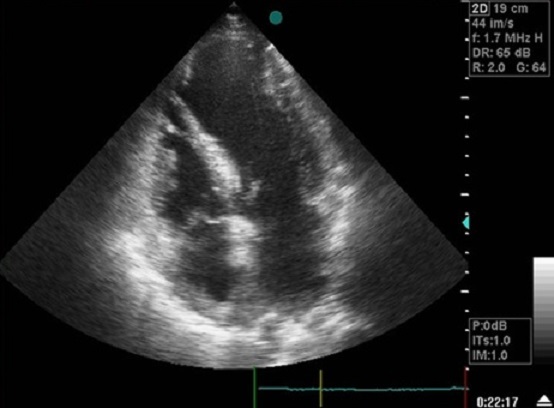
Coupe 4 cavités à l'ETT montrant une CIV apicale

**Figure 2:**
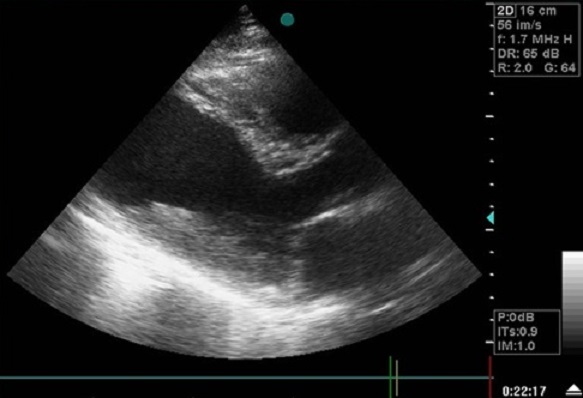
Coupe parasternal grand axe à l'ETT montrant un anévrysme ventriculaire gauche

**Figure 3:**
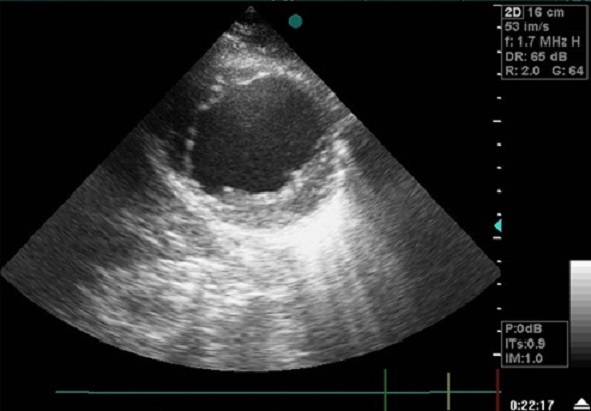
Coupe parasternal petit axe à l'ETT montrant un volumineux anévrysme du VG

La radiographie thoracique a retrouvé une cardiomégalie avec un index cardiothoracique à 60% et un arc inferieur gauche globuleux avec une pointe sous diaphragmatique. L’électrocardiogramme s'est inscrit en rythme régulier sinusal avec des ondes Q de nécrose en antérieur. L’échocardiographie transthoracique a objectivé une akinésie apicale et inféro-septale du VG avec une dysfonction systolique du ventricule gauche (fraction d’éjection estimée à 38%); ainsi qu'une CIV musculaire restrictive de 18mm au niveau du segment apical de la paroi inféro-septale partiellement colmatée par les trabéculations du ventricule droit (flux estimé à 4,18m/s) (Figure 1), et un large anévrysme apical du VG mesurant environ 60mm de diamètre longitudinal (Figure 2, Figure 3), avec des pressions de remplissage ventriculaire gauche élevées. La coronarographie a retrouvé des sténoses étagées du segment moyen de l'artère inter ventriculaire antérieure(IVA), une occlusion du segment apical de l'IVA, ainsi que des sténoses étagées et serrées de la seconde portion de la coronaire droite.

Le patient a été opéré en urgence en raison de l'instabilité hémodynamique. L'induction anesthésique a été réalisée sous support isotrope positif à base de dobutamine à raison de 10gamma/kg. min et d'adrénaline à la dose de 0. 02gamma/kg. min, ainsi qu'une assistance circulatoire par ballon de contre pulsion intra-aortique (BCPIA). La voie d'abord a été une sterno-péricardotomie médiane verticale avec abord de la jambe droite pour prélèvement de la veine saphène interne. La circulation extracorporelle(CEC) a été menée en hypothermie modérée à 32°C et hémodilution partielle, entre une canule aortique au pied du tronc artériel brachio-céphalique et deux canules caves ainsi qu'une canule de décharge ventriculaire gauche via la veine pulmonaire supérieure droite. La protection myocardique a été assurée par une cardioplégie cristalloïde antérograde. La durée de CEC était de 220min et celle du clampage aortique était de 114 min.

**Figure 4:**
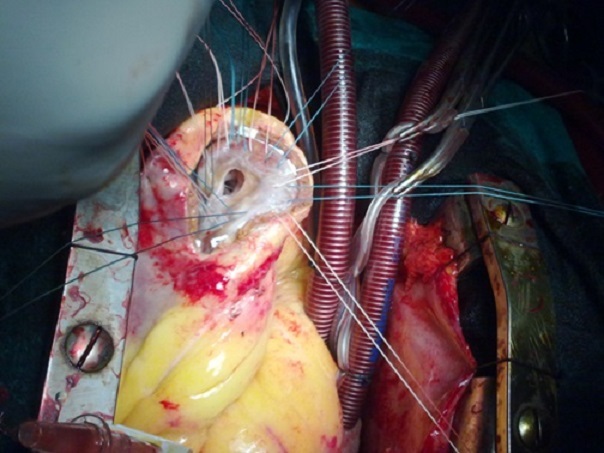
Vue opératoire: exposition d'une CIV apicale après ventriculotomie gauche

**Figure 5:**
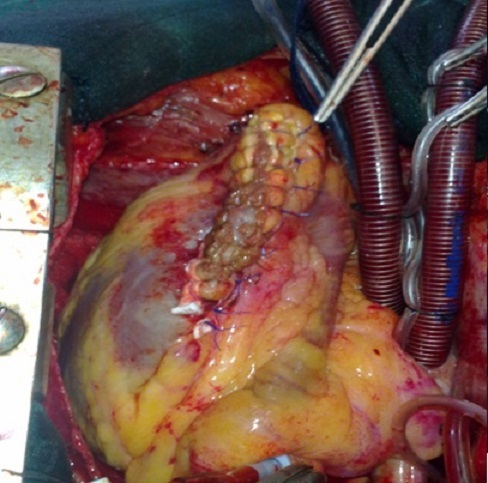
Vue opératoire: fermeture de l'anévrysme du VG sur 2 attelles en téflon renforcé par un sujet continu

La correction chirurgicale a consisté en une ventriculotomie de 7 cm au milieu de la zone anévrysmale parallèle à l'IVA, avec résection des tissus nécrosés. On retrouve une CIV apicale avec des bords friables et nécrosés, qui a été fermée par un patch en dacron cousu par une couronne de points en U patchés (Figure 4). En raison du caractère oblongue de l'anévrysme du VG, de la taille assez généreuse de la cavité résiduelle et de la situation des piliers mitraux, on a opté pour la technique de résection linéaire avec fermeture directe des berges anévrysmales par des points séparés en U appuyés sur 2 attelles en téflon renforcée par un double surjet d'hémostase au fil monobrin (Figure 5), avec un encollage des sutures. Par la suite, on a réalisé un pontage aorto-coronaire sur la coronaire droite par une veine saphène interne, tandis que l'IVA était prise dans l'anévrysme.

**Figure 6:**
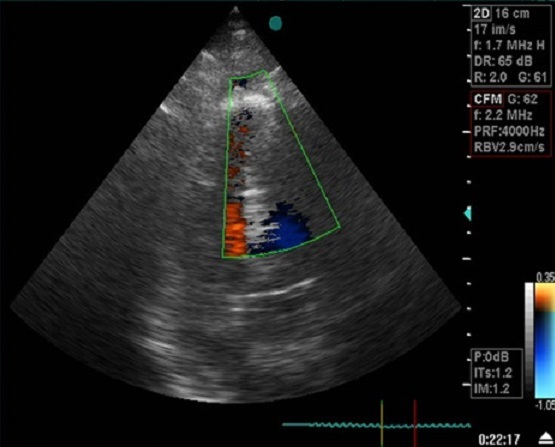
ETT de contrôle: coupe apicale 4 cavités montrant la fermeture de la CIV

La sortie de CEC était difficile et les suites opératoires ont été marquées par la survenue d'un état de choc hémodynamique ayant nécessité le maintien d'une assistance mécanique par BCPIA et d'un support isotrope positif par dobutamine (15gamma/kg. min),adénaline(0. 08gamma/kg. min) et noradrénaline(0. 5ug/kg. min). Le séjour en réanimation a duré dix jours Le contrôle échocardiographique s'est révélé tout à fait satisfaisant avec un patch de CIV étanche et des pressions de remplissage qui se sont normalisées (Figure 6).

## Discussion

Les complications de l'IDM sont nombreuses et constituent toute la gravité de la maladie coronaire. L'association d'une CIV et d'un anévrysme ventriculaire gauche, retrouvée chez notre patient, constitue une complication rarissime et fortement létale nécessitant une prise en charge chirurgicale urgente [1]. L'incidence de cette complication a été évaluée entre 1% et 2% des IDM, mais elle est responsable de 5% des décès en phase aiguë d'infarctus [1]. Son incidence actuelle est moindre (0,2%) dans l’étude GUSTO-I étudiant le bénéfice d'une thrombolyse précoce dans les IDM aigus [2]. Les CIV peuvent apparaître dans un délai de quelques heures à une semaine après la nécrose, avec une majorité entre le deuxième et quatrième jours [2, 3]. Ce délai est, néanmoins, de cinq semaines chez notre patient.

L’échocardiographie transthoracique est l'examen de choix dans le diagnostic et l’évaluation des CIV post infarctus avec une sensibilité et une spécificité très élevées [4]. Sa prise en charge est chirurgicale, avec la difficulté de réparation à partir de tissus infarcis fragiles. Deux attitudes sont généralement adoptées: une réparation différée de 6 à 8 semaines après l'IDM afin de réaliser une réparation sur des tissus cicatriciels plus solides et ce en cas de stabilité hémodynamique et de CIV de petite taille; ou encore, une réparation en urgence du fait de l'instabilité hémodynamique du patient. [5] Selon les recommandations de l'American College of Cardiology'American Heart Association (ACC-AHA, recommandation classe I) [5], la réparation chirurgicale ne doit pas être différée, quel que soit l’état clinique du patient.

Malgré une prise en charge adéquate, le pronostic de cette pathologie est sombre, avec une mortalité estimée à 30%. Les facteurs pronostiques sont principalement représentés par la pression artérielle systémique, la pression de l'oreillette droite et la durée de circulation extracorporelle(CEC). [6] Les premières interventions d'un anévrisme ventriculaire ont été décrites en 1944 par Beck [7], qui réalisait un renforcement externe de la paroi anévrismale par l'aponévrose du fascia lata. La première réparation d'un anévrisme par suture linéaire directe sous CEC est décrite par Cooley et al. [8] en 1958. Ce sont les travaux de Dor et al. [9] et Jatene [10] sur l'importance de conserver une cinétique et une géométrie ventriculaires elliptiques qui ont permis de développer la réparation du VG. L'incidence des anévrismes du VG est estimée à 7,6% dans l’étude CASS [11]. Elle est certainement moindre à l'heure actuelle grâce à la reperfusion précoce des infarctus (thrombolyse, angioplastie) et l'utilisation des inhibiteurs de l'enzyme de conversion, qui agissent respectivement sur la phase initiale de formation anévrismale et le remodelage tardif post infarctus du VG.

En ce qui concerne la revascularisation myocardique associée à la cure chirurgicale de la complication de l'IDM, les avis sont partagés. Les pontages associés à une chirurgie en urgence pour CIV et anévrysme du VG n'augmentent pas la morbidité et la mortalité hospitalières [12–14]. En revanche, certains auteurs ne préconisent pas cette chirurgie associée du fait de l'absence de bénéfice à moyen terme [13] alors que d'autres rapportent une amélioration de la survie à moyen terme [12, 14]. Notre patient est unique en ce sens qu'il a présenté une double complication mécanique de l'IDM cinq semaines après l’épisode inaugural sans aucune preuve d'une récidive infarctoide.

## Conclusion

L'association d'une CIV et d'un infarctus du VG est une entité rare et se produit habituellement dans les deux semaines qui suivent un IDM. Cependant, et ce qui est le cas de notre patient, ces complications peuvent survenir plusieurs semaines après. Un diagnostic rapide et une prise en charge chirurgicale urgente améliore considérablement le pronostic du patient.
